# Devascularization and Transection Procedures

**DOI:** 10.1155/1991/36854

**Published:** 1991

**Authors:** Yasuo Idezuki

**Affiliations:** Second Department of Surgery University of Tokyo Faculty of Medicine Tokyo Japan

## Abstract

Transection and devascularization procedures have been by far the most popular operations for the treatment of oesophagogastric varices in Japan during the last two decades^1-5^ and although since the new development of endoscopic sclerotherapy during the last ten years the number of patients treated by operation has decreased considerably, these procedures still remain the treatment of choice in many of the surgical institutions in Japan, especially in good-risk patients with oesophagogastric varices.


*HPB Surgery*, 1991. Vol. 4, pp 33–38
Reprints available directly from the publisher
Photocopying permitted by license only

© 1991 Harwood Academic Publishers GmbH
Printed in the United Kingdom

### DEVASCULARIZATION AND TRANSECTION PROCEDURES

YASUO IDEZUKI
*Second Department of Surgery, University of Tokyo, Faculty of Medicine, Tokyo, Japan*

*(Received 17 September 1990)*

Transection and devascularization procedures have been by far the most popular operations for the treatment of oesophagogastric varices in Japan during the last two decades[1–5] and although since the new development of endoscopic sclerotherapy during the last ten years the number of patients treated by operation has decreased considerably, these procedures still remain the treatment of choice in many of the surgical institutions in Japan, especially in good-risk patients with oesophagogastric varices.

KEY WORDS: Portal hypertension, devascularization, transection

### OUR EXPERIENCE

During the period January, 1964 to March, 1990, 532 patients have been treated by transection and devascularization procedures in our department: diseases causing portal hypertension and varices were liver cirrhosis in 381, idiopathic portal hypertension (IPH) 104, extrahepatic portal obstruction (EHPO) in 38, schistosomiasis japonica in 4, Budd-Chiari syndrome in 2, and other diseases in 3. Approximately 85% of cirrhotic patients were either cryptogenic or posthepatitic. Mean ages of these patients were 48.6 ± 10.9 years in cirrhosis, 48.2 ± 12.0 years in IPH, and 23.4 ± 15.8 years in EHPO (Mean ± SD). Male: female ratio was approximately 2:1. Results of non-shunting operations in patients with cirrhosis, IPH and EHPO were analysed. Overall operative mortality was 5.0%, it was observed only in patients with liver cirrhosis and was higher in emergency cases (23.3%) compared to elective (3.6%) or prophylactic(3.9%) cases.

Operative mortality was also high in Child C patients (17.1%) compared to Child A and B patients (0% and 2.3% respectively). Long-term results in these patients differed significantly by the nature of original diseases and severity of hepatic deterioration. Cumulative survival rates at 5 years in patients with EHPO were 97.3%, 92.0% in IPH, and 62.4% in cirrhosis, and those at 10 years were 90.6%, 76.7%, and 32.0% respectively. Cumulative survival rates at 5 years in patients with cirrhosis classified as Child A was 76.6%, 71.6% in Child B, and 37.4% in Child C. Cumulative survival rates at 10 years were 54.9% in Child A, 35.5% in Child B and 13.0% in Child C.

Correspondence to: Prof. Yasuo Idezuki, Second Department of Surgery, University of Tokyo, Faculty of Medicine,, Tokyo, Japan

Recurrent bleeding rate of non-shunting operations (including both bleeding from recurrent varices and bleeding from peptic ulcers and hemorrhagic gastritis) was around 20% at 10 years, irrespective of the types of operations. Postoperative follow-up by endoscopy revealed that recurrence rate of varices after operation was 5% at one year, 16% at 2 years, 26% at 3 years, 30% at 4 years and 35% at 5 years. Recurrence of varices was observed more often in patients with alcoholic cirrhosis or in those with cirrhosis complicated by hepatocellular carcinoma. Recently, recurrence of varices after these non-shunting operations have been successfully treated by endoscopic sclerotherapy. Usually a session or two of endoscopic injection sclerotherapy could erradicate the residual or recurrent varices. Our experiences have clearly shown that devascularization and transection procedures are a safe and preferable operation in patients in Child A and B categories, and also feasible as an emergency operation in these cases when necessary. However, these operations are not indicated in Child C patients[6]. In assessing a patient's risk for these procedures, albumin and bilirubin levels in serum, S-GOT, S-GPT, antipyrin clearance, ICG–15, K–ICG, prothrombin time and hepaplastin test are very informative (Table 1).

**Table 1** Indications for surgical treatment have become more strict since 1986 when technique of sclerotherapy was established.

### Indications for Surgical Treatment

| | before 1986 | after 1986 |
|---|---|---|
| Age | 70 year old> | 65 year old> |
| History | history of bleeding | [ditto] |
| Endoscopic Findings | R-C sign(+), $F_{2-3}$, $C_B$ | |
| Clinical Symptoms | Encephalopathy (−)<br>Ascites (−)<br>Cachexia (−) | [ditto] |
| Liver Function Tests | | |
| Albumin | 2.8 g/dl< | 3.0 g/dl< |
| Bilirubin | 3.5 mg/dl> | 2.0 mg/dl> |
| S-GOT | 200 units> | 100 units> |
| S-GPT | 200 units> | 100 units> |
| Antipyrin Clearance | 0.10 ml/min/kg< | [ditto] |
| Prothrombin Time | 50 %< | [ditto] |
| Hepaplastin Test | 50 %< | [ditto] |
| ICG R-15 | 40 %> | [ditto] |
| K-ICG | $0.04\ min^{-1}$< | $0.06\ min^{-1}$< |
| | No severe complications in other organs | [ditto] |

### ENDOSCOPIC SCLEROTHERAPY

Endoscopic sclerotherapy has become very popular in Japan during the last several years, and now the number of patients treated by this modality of treatment exceeds that of those treated by operation[7]. A recent nation-wide survey revealed that approximately 85% of patients with varices were treated initially by sclerotherapy, and only 15% treated by surgery. Reasons for this drastic change are not clear, however the following may explain some of the reasons: improvement of technical skills and equipments of endoscopy, development of effective sclerosants, increasing number of aged patients, increasing incidence of hepatocellular carcinoma in post-hepatitic cirrhosis, increasing number of endoscopists in Japan and their interests and enthusiasm for this modality of treatment.

We started sclerotherapy for varices in 1969 in our department and treated 169 patients in whom surgery was not indicated, namely in Child C patients, patients complicated with unresectable hepatoma, patients with residual or recurrent varices after operation, rejection of operation by patient, or in whom treatment was performed as a prophylactic measure.

Cumulative survival rates of these patients were 72% at one year, 45% at 3 years, and 34% at 5 years. Cumulative survival rate in Child A patients was 72.0% which was not significantly different from that of non-shunting operations, but those of Child B and C patients were much lower than those of patients treated by surgery (Figures 1,2,3). This was partly because the sclerotherapy treated group included

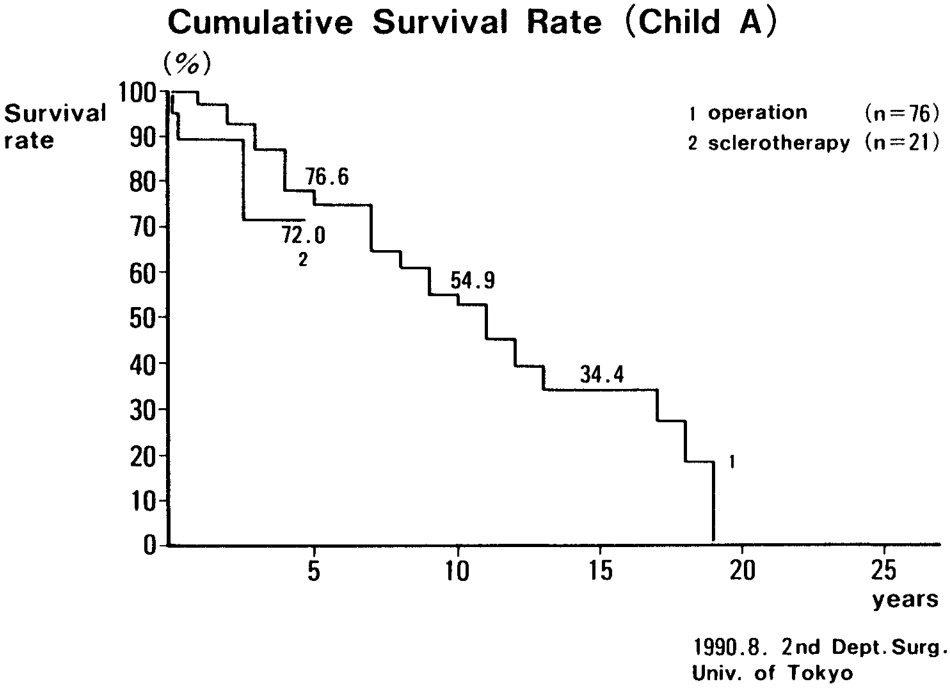

**Figure 1** Cumulative survival rate in Child A patients; Non-shunting operation vs sclerotherapy

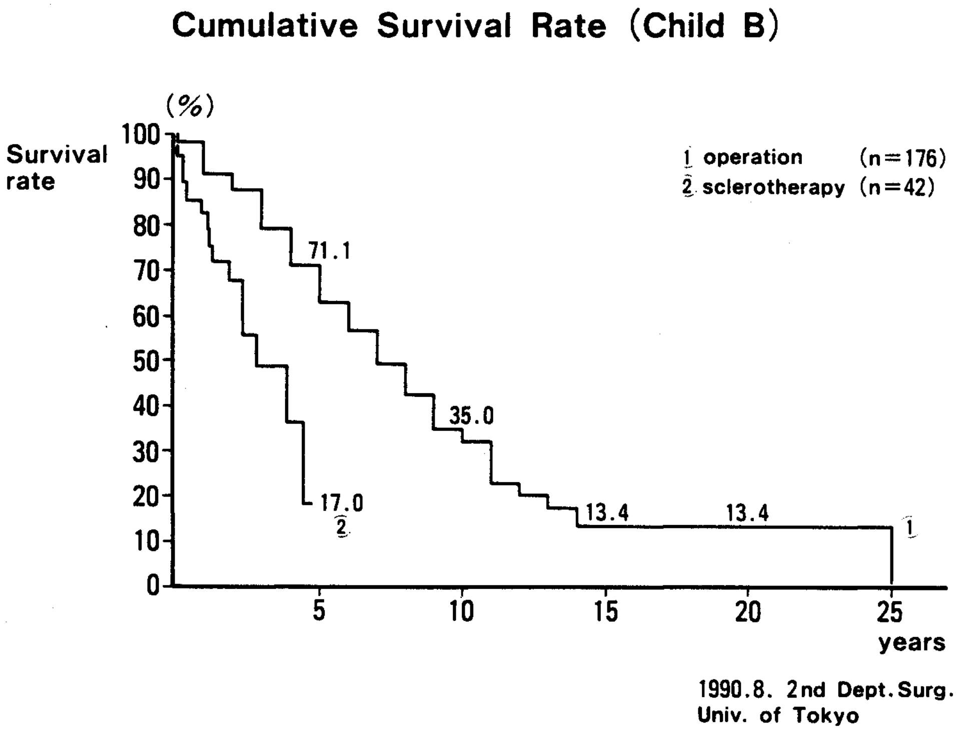

**Figure 2** Cumulative survival rate in Child B patients; Non-shunting operation vs sclerotherapy.

more patients who were complicated by hepatoma. Recurrence rate of bleeding after sclerotherapy was rather high, 22% of patients had at least one episode of rebleeding by the end of one year after sclerotherapy, and rebleeding rate reached up to 33% at 3 years. Oesophageal varices in Child C patients did not improve even after several sessions of sclerotherapy and patients with large caliber varices with increased blood flow were difficult to treat by sclerotherapy, who were eventually treated by surgery. It is rather disappointing for us surgeons that sclerotherapy has not been successful in many of the poor risk patients in whom operation can not be tolerated.

### PROPHYLACTIC TREATMENT OF OESOPHAGOGASTRIC VARICES

Prophylactic treatment of oesophagogastric varices has been a target of controversy among surgeons for many years, since predictability of rupture of varices has been doubted.

Endoscopic findings, and intravariceal pressure are considered to be of significant value in predicting variceal rupture[8–11].

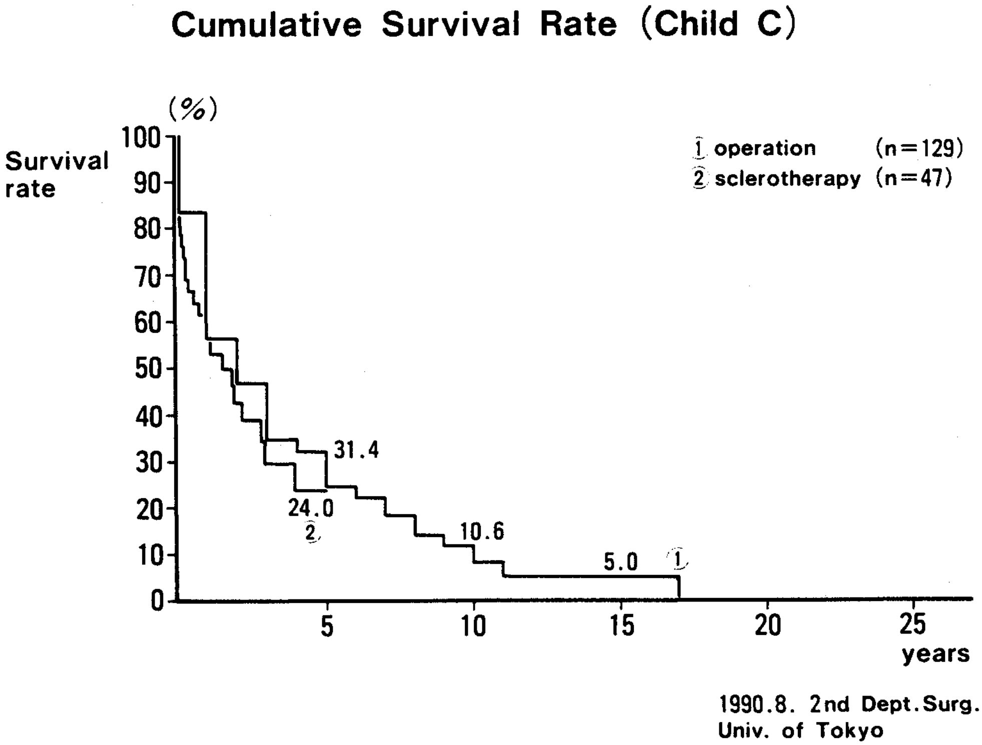

**Figure 3** Cumulative survival rate in Child C patients; Non-shunting operation vs sclerotherapy.

Correlation between Red-Color signs on endoscopy and intravariceal pressure was also found. On the basis of Red-Color signs and prognostic blue color of varices by endoscopy, prophylactic operation and prophylactic sclerotherapy have been widely performed in Japan[7]. Only few prospective controlled studies of prophylactic operations have been reported so far[12]. Mortality rate and morbidity directly related to prophylactic treatment have been very low with non-shunting operations and sclerotherapy.

Reports of prophylactic controlled studies with sclerotherapy are accumulating,[13–19] but the results have not been unanimous, and therefore not convincing.

Further study seems to be necessary before prophylactic treatment of oesophageal varices can be justified.

### *References*

1. Idezuki, Y., Sugiura, M. and Sakamoto, K. *et al.* (1967) Rationale for transthoracic esophageal transection for bleeding varices. *Diseases of Chest*, **52**, 621–631
2. Sugiura, M. and Futagawa, S. (1973) A new technique for treating esophageal varices. *Journal of Thoracic and Cardiovascular Surgery*, **66**, 667–685

3. Hassab, M.A. (1967) Gastroesophageal decongestion and splenectomy in the treatment ot esophageal varices in bilharzial cirrhosis. Further studies with a report on 355 operations. *Surgery*, **61**, 169–176
4. Yamamoto, S., Hidemura, R. and Sawada, M. *et al.* (1976) The results of terminal esophago-proximal gastrectomy (TEPG) with extensive devascularization and splenectomy for bleeding esophageal varices in cirrhosis. *Surgery*, **80**, 106–114
5. Inokuchi,, K. (1985) Present status of surgical treatment of esophageal varices in Japan: a nation-wide survey of 3588 patients. *World Journal of Surgery*, **9**, 171–180
6. Idezuki, Y. and Snjo K. (1989) Twenty-five-year experiences with esophageal transection for esophageal varices. *Journal of Thoracic and Cardiovascular Surgery*, **98**, 876–883
7. Idezuki, Y. (1988) Current status of treatment of esophageal varices in Japan: endoscopic sclerotherapy in Japan. In Treatment of esophageal varices (Idezuki, Y. editor) *Excerpta Medica*, Amsterdam, pp 367–374
8. Japanese Research Society for Portal Hypertension. (1980) General rules for recording endoscopic findings on esophageal varices. *Japanese Journal of Surgery*, **10**, 84–87
9. Beppu, K., Inokuchi, K. Koyanagi, N. *et al.* (1981) Prediction of variceal hemorrhage by esophageal endoscopy. *Gastrointestinal Endoscopy*, **27**, 213–218
10. Koyanagi, N. and Cooperative study group of portal hypertension in Japan. (1988) Prognostic blue varices as a discriminant factor of findings of esophageal varices. *Japanese Journal of Surgery*, **18**, 142–145
11. Paquet, K.J. (1988) Indications and early and long-term results of paravariceal immediate, elective, and prophylactic injection sclerotherapy. In Treatment of Esophageal varices (Idezuki Y, editor) *Excerpta Medica*, Amsterdam pp 1–22
12. Inokuchi, K. and Cooperative study group of portal hypertension in Japan. (1984) Prophylactic portal non-decompression surgery in patients with esophageal varices – An interim report. *Annals of Surgery*, **200**, 61–65
13. Terblanche J., Bornman, P.C., Kahn, D. *et al* (1983) Failure of repeated injection sclerotherapy to improve long-term survival after oesophageal variceal bleeding. A five year prospective controlled clinical trial. *Lancet*, **2**, 1328–1332
14. Paquet, K.J. (1982) Prophylactic endoscopic sclerosant treatment of esophageal wall in varices — a prospective controlled randomized trial. *Endoscopy*, **14**, 4–7
15. Potzi, R., Bauer, P. and Reichel, W. *et al.* (1989) Prophylactic endoscopic sclerotherapy of oesophageal varices in live cirrhosis. A multicentre prospective controlled randomized trial in Vienna. *Gut*, **30**, 873–879
16. Sauerbruch, T., Wotzka, R. and Koepcke, W. *et al.* (1988) Prophylactic sclerotherapy before the first episode of variceal hemorrhage in patients with cirrhosis. *New England Journal of Medicine*, **319**, 8–15
17. Witzel, L., Wolbergsd, E. and Merki, H. (1985) Prophylactic endoscopic sclerotherapy of oesophageal varices. *Lancet*, **2**, 773–775
18. Santangelo, W.C., Dueno, M.I. and Estes, B.L. *et al.* (1988) Prophylactic sclerotherapy of large esophageal varices. *New England Journal of Medicine*, **318**, 814–818
19. Piai, G., Cipolletta, L., Claar, M. *et al.* (1988) Prophylactic sclerotherapy of high-risk esophageal varices: Results of multicentric prospective controlled trial. *Hepatology*, **8**, 1495–1500

